# Supplementary material for: Heart murmur detection from phonocardiogram recordings: The George B. Moody PhysioNet Challenge 2022
Source: PLOS Digit Health. 2023 Sep 11;2(9):e0000324. doi: 10.1371/journal.pdig.0000324 (PMC10495026; doi:10.1371/journal.pdig.0000324)
Supplement: S1 Appendix — (PDF) [file pdig.0000324.s001.pdf]

Heart murmur detection from phonocardiogram recordings: The George B. Moody PhysioNet Challenge 2022

Matthew A. Reyna[1*], Yashar Kiarashi[1], Andoni Elola[2], Jorge Oliveira[3], Francesco Renna[4], Annie Gu[1], Erick A. Perez Alday[1], Nadi Sadr[1,5], Ashish Sharma[1†], Jacques Kpodonu[6], Sandra Mattos[7], Miguel T. Coimbra[4], Reza Sameni[1], Ali Bahrami Rad[1], Gari D. Clifford[1,8]

1 Department of Biomedical Informatics, Emory University, Atlanta, GA, USA
2 Department of Electronic Technology, University of the Basque Country UPV/EHU, Eibar, Gipuzkoa, Spain
3 REMIT, Universidade Portucalense, Porto, Portugal
4 INESC TEC, Faculdade de Ciências, Universidade do Porto, Porto, Portugal
5 ResMed, Sydney, Australia
6 Division of Cardiac Surgery, Beth Israel Deaconess Medical Center, Harvard Medical School, Boston, MA, USA
7 Unidade de Cardiologia e Medicina Fetal, Real Hospital Português, Recife, Pernambuco, Brazil
8 Department of Biomedical Engineering, Emory University and the Georgia Institute of Technology, Atlanta, GA, USA

¤Current Address: Department of Biomedical Informatics, Emory University, Atlanta, Georgia, United States
†Deceased
* matthew.a.reyna@emory.edu

S1 Appendix. Additional scoring metrics

We defined additional scoring metrics to allow us to make more direct comparisons between methods and tasks.

In particular, we defined a weighted accuracy metric for the clinical outcome identification task as

$$a_{\text{outcome}} = \frac{5n_{\text{TP}} + n_{\text{TN}}}{5(n_{\text{TP}} + n_{\text{FN}}) + (n_{\text{FP}} + n_{\text{TN}})}, \tag{1}$$

where Table 2 defines a two-by-two confusion matrix $N = [n_{ij}]$ for the clinical outcome abnormal and normal classes.

We also defined the total cost of diagnosis and treatment with algorithmic pre-screening of murmurs as

$$\begin{aligned} c_{\text{outcome}}^{\text{total}} = &f_{\text{algorithm}}(n_{\text{patients}}) \\ &+ f_{\text{expert}}(o_{\text{PA}} + o_{\text{PN}} + o_{\text{UA}} + o_{\text{UN}},\, n_{\text{patients}}) \\ &+ f_{\text{treatment}}(o_{\text{PA}} + o_{\text{UA}}) \\ &+ f_{\text{error}}(o_{\text{AA}}), \end{aligned} \tag{2}$$

where Table A1 defines a three-by-two confusion matrix $O = [o_{ij}]$ for the clinical outcome abnormal and normal classes, n_{patients} is the total number of patients, and $f_{\text{algorithm}}$, f_{expert}, $f_{\text{treatment}}$, f_{error} are defined above.

		Clinical Outcome Expert	
		Abnormal	Normal
Murmur Classifier	Present	o_{PA}	o_{PN}
	Unknown	o_{UA}	o_{UN}
	Absent	o_{AA}	o_{AN}

Table A1. Confusion matrix for murmur detection task with three classes (murmur present, murmur unknown, and murmur absent) using clinical outcomes with two classes (clinical outcome abnormal and clinical outcome normal). The columns are the ground truth labels from the human annotator, and the rows are the classifier outputs. The entries of the confusion matrix provide the number of patients with each classifier output for each ground truth class.
