# Supplementary material for: Heart murmur detection from phonocardiogram recordings: The George B. Moody PhysioNet Challenge 2022
Source: PLOS Digit Health. 2023 Sep 11;2(9):e0000324. doi: 10.1371/journal.pdig.0000324 (PMC10495026; doi:10.1371/journal.pdig.0000324)
Supplement: S2 Appendix — (PDF) [file pdig.0000324.s002.pdf]

Heart murmur detection from phonocardiogram recordings: The George B. Moody PhysioNet Challenge 2022

Matthew A. Reyna[1*], Yashar Kiarashi[1], Andoni Elola[2], Jorge Oliveira[3], Francesco Renna[4], Annie Gu[1], Erick A. Perez Alday[1], Nadi Sadr[1,5], Ashish Sharma[1†], Jacques Kpodonu[6], Sandra Mattos[7], Miguel T. Coimbra[4], Reza Sameni[1], Ali Bahrami Rad[1], Gari D. Clifford[1,8]

1 Department of Biomedical Informatics, Emory University, Atlanta, GA, USA
2 Department of Electronic Technology, University of the Basque Country UPV/EHU, Eibar, Gipuzkoa, Spain
3 REMIT, Universidade Portucalense, Porto, Portugal
4 INESC TEC, Faculdade de Ciências, Universidade do Porto, Porto, Portugal
5 ResMed, Sydney, Australia
6 Division of Cardiac Surgery, Beth Israel Deaconess Medical Center, Harvard Medical School, Boston, MA, USA
7 Unidade de Cardiologia e Medicina Fetal, Real Hospital Português, Recife, Pernambuco, Brazil
8 Department of Biomedical Engineering, Emory University and the Georgia Institute of Technology, Atlanta, GA, USA

¤Current Address: Department of Biomedical Informatics, Emory University, Atlanta, Georgia, United States
†Deceased
* matthew.a.reyna@emory.edu

S2 Appendix. Mathematical derivation of the cost-based scoring metric

We defined the cost of expert screening to reflect the non-linear costs associated with a healthcare system with a limited screening capacity. While providing fewer screenings incurs a lower total screening cost, the screenings are typically more expensive on a per-screening basis because of the underutilized capacity of the system. Similarly, while more screenings incur higher costs, the screenings are also typically more expensive on a per-screening basis because of the inadequate capacity of the system.

Let s be the number of expert screenings in a patient cohort of t patients, and let $x = s/t$ be the fraction of the cohort receiving expert screenings. We defined $g_{\text{expert}}(x) = a + bx + cx^2 + dx^4$ as the mean expert screening cost for screening a fraction x of a cohort, and we in turn defined $f_{\text{expert}}(s, t) = g_{\text{expert}}(s/t)t = at + bs + \frac{cs^2}{t} + \frac{ds^4}{t^3}$ as the total cost for s expert screenings in a cohort of t patients. These quantities are quartic functions with four unknowns, allowing us to satisfy four criteria:

1. We set $g_{\text{expert}}(0) = 25$ to define a cost for maintaining the ability to perform expert screening, even when screening $x = 0$ of a cohort, i.e., screening none of the cohort.

2. We set $\frac{d}{dx}\, g_{\text{expert}}(x)/x\,|_{x=\frac{1}{4}} = 0$ so that mean expert screening cost cost achieved its minimum when screening $x = \frac{1}{4}$ of a cohort, which was roughly half of the prevalence rate of abnormal cases in the database,

3. We set $g_{\text{expert}}(\frac{1}{2}) = 1000$ so that the mean expert screening cost was \$1000 when screening $x = \frac{1}{2}$ of a cohort, which is roughly the prevalence rate of abnormal cases in the database.
4. We set $g_{\text{expert}}(1) = 10000$ so that the mean expert screening cost was \$10000 when screening $x = 1$, i.e., screening all of the cohort, which is ten times the cost of screening half of the database.

The unique coefficients that satisfy these conditions are $a = 25$, $b = 397$, $c = -1718$, and $d = 11296$.
