# Supplementary material for: Heart murmur detection from phonocardiogram recordings: The George B. Moody PhysioNet Challenge 2022
Source: PLOS Digit Health. 2023 Sep 11;2(9):e0000324. doi: 10.1371/journal.pdig.0000324 (PMC10495026; doi:10.1371/journal.pdig.0000324)
Supplement: Attachment — Submitted filename: PDIG-D-23-00131_comments.pdf [file pdig.0000324.s003.pdf]

Heart murmur detection from phonocardiogram recordings

The manuscript presents results from the “Heart murmur detection from phonocardiogram recordings: The George B. Moody PhysioNet Challenge 2022”. It is well written and easy to follow. The manuscript uses up to date references; which are all well linked within the document. All reference materials have been provided.

However, the following could be addressed.

- The abstract is great, but consider adding a paragraph about the results (submitted algorithms) from the challenge. The abstract presents the background information; and how the challenge was conducted, but is very silent about the results (which algorithm performed best from the challenge). You might also consider mentioning the aim of this opinion paper in the abstract, to provide a reader with the general picture of the paper.
- The usage of the phrase “This year’s challenge” appears misleading in some paragraphs. May be consider using “The 2022 year’s challenge”; so that it is easy for the reader not to confuse it with the current year.
- The paragraph “while algorithms that did not learn from the training set performed the same or better” on page 12/24 is incomplete.
- The paragraph “requires monitoring and/or intervention” on page 14/24 is incomplete.
- Check paragraph “models and k = 2 for the RF models.” on page 13/24
