# Supplementary material for: Heart murmur detection from phonocardiogram recordings: The George B. Moody PhysioNet Challenge 2022
Source: PLOS Digit Health. 2023 Sep 11;2(9):e0000324. doi: 10.1371/journal.pdig.0000324 (PMC10495026; doi:10.1371/journal.pdig.0000324)
Supplement: Attachment — Submitted filename: 2022 Challenge Manuscript Review Response.pdf [file pdig.0000324.s004.pdf]

We thank the reviewers for their generous feedback on our manuscript. We have responded to each point in their reports, and we have updated the manuscript as described below.

The reviewer comments are in **blue**. Our responses are in **black**.

Reviewer #1: The manuscript presents results from the “Heart murmur detection from phonocardiogram recordings: The George B. Moody PhysioNet Challenge 2022”. It is well written and easy to follow. The manuscript uses up to date references; which are all well linked within the document. All reference materials have been provided.

Thank you for the kind comments.

However, the following could be addressed.

- The abstract is great, but consider adding a paragraph about the results (submitted algorithms) from the challenge. The abstract presents the background information; and how the challenge was conducted, but is very silent about the results (which algorithm performed best from the challenge). You might also consider mentioning the aim of this opinion paper in the abstract, to provide a reader with the general picture of the paper.

The third paragraph of the abstract describes the Challenge algorithms. Following this feedback, we have expanded this paragraph from

> We received 779 algorithms from 87 teams during the course of the Challenge, resulting in 53 working codebases for detecting heart murmurs and abnormal cardiac function from PCGs. These algorithms represent a diversity of approaches from both academia and industry.

to

> We received 779 algorithms from 87 teams during the Challenge, resulting in 53 working codebases for detecting heart murmurs and abnormal cardiac function from PCG recordings. These algorithms represent a diversity of approaches from both academia and industry, including methods that use more traditional machine learning techniques with engineered clinical and statistical features as well as methods that rely primarily on deep learning models to discover informative features.

We intentionally did not emphasize which algorithm performed the best. There are two different tasks for the Challenge with two different scoring metrics and sets of labels. We explore these details in the manuscript, where there is more space.

- The usage of the phrase “This year’s challenge” appears misleading in some paragraphs. May be consider using “The 2022 year’s challenge”; so that it is easy for the reader not to confuse it with the current year.

We agree. We have updated the manuscript to replace each instance of “this year’s Challenge” with “the George B. Moody PhysioNet Challenge 2022” or the “2022 Challenge”.

- The paragraph “while algorithms that did not learn from the training set performed the same or better” on page 12/24 is incomplete.

We have expanded the entire this sentence from

> Algorithms that truly learned from the training data performed much worse when retrained on the modified training set, while algorithms that did not learn from the training set performed the same or better.

to

> Algorithms that truly learned from the training data performed much worse when retrained on the modified training set, but algorithms that did not learn from the training set performed the same or better on the modified training set.

The full paragraph is now

> To assess the robustness of the algorithms, we tested whether they actually learned from the training set; algorithms that cannot learn from new data have more limited use. To do so, we trained them on a subset of the training set with permuted labels and ran the retrained models on the validation and test sets with the original labels; we did not change any of the publicly available training data or labels but simply the size of the training set and the relationship between the data and the labels in the training set. Algorithms that truly learned from the training data performed much worse when retrained on the modified training set, but algorithms that did not learn from the training set performed the same or better on the modified training set. Only 35 of the 53 working entries were robust enough for us to complete this process. The remaining 18 working entries either crashed or achieved the same or a better score when retrained on the modified training set. While we encouraged teams to submit robust code, we did not share the details about how we would test the robustness of their algorithms to encourage them to make their code as robust in as many ways as possible. Therefore, since we did not provide advance notice of the exact requirements for robustness, we did not disqualify teams whose algorithms failed this test.

- The paragraph “requires monitoring and/or intervention” on page 14/24 is incomplete.

We have changed this paragraph from

> Some heart murmurs are pathological, indicating a physiological problem that requires monitoring and/or intervention. Other heart murmurs are innocent. The human annotator did not identify which heart murmurs were pathological or innocent; the full examination

is the only evidence in the dataset that helps to characterize the severity of any of the heart murmurs. We only evaluated the ability of algorithms to identify heart murmurs, and not their ability to identify pathological heart murmurs, but such as [sic] task is still valuable for screening."

to

Some heart murmurs are pathological, indicating a physiological problem that requires monitoring and/or intervention. Other heart murmurs are innocent, with sounds resulting from normal blood flow, and do not require treatment. However, the human annotator did not identify which heart murmurs were pathological or innocent; the clinical outcomes, which reflect a full examination of the patient, are the strongest evidence in the dataset for characterizing the severity of any heart murmurs or underlying cardiac disease. Therefore, we only evaluated the ability of algorithms to identify heart murmurs, not their ability to identify pathological heart murmurs, but such a task is still valuable for screening for cardiovascular disease.

- Check paragraph "models and k = 2 for the RF models." on page 13/24

We checked this paragraph, and we made minor edits to this section to improve clarity. In general, we took the opportunity to reread the entire manuscript and make minor edits to improve clarity whenever possible; all such edits are shown in the "track changes" version of the manuscript.

Reviewer #2: Thank you for the opportunity to review this interesting manuscript. This purpose of the paper was to report findings from The 2022 George B. Moody PhysioNet Challenge (formerly the PhysioNet/Computing in Cardiology Challenge). This challenge invited teams to develop fully automated approaches for detecting heart murmurs and abnormal cardiac function from PCG recordings. The investigators sourced and shared PCG recordings for up to four auscultation locations from a largely pediatric population in Brazil, and teams were asked to identify both heart murmurs and the clinical outcomes of a full diagnostic screening from the recordings. The Challenge explored the diagnostic potential of algorithmic approaches for interpreting PCG recordings. Below are suggestions designed to enhance the readability, clinical understanding and reproducibility of this already well written manuscript.

The format of the paper made it much easier to review this manuscript – thank you.

Thank you, too, for the kind comments.

Title – consider adding "Pediatric" to the title.

The patient population is primarily pediatric, but it also includes pregnant adults. It does not include the fetuses of the pregnant patients. We considered adding "Pediatric" to the title, but we

were concerned that it may mislead readers about the patient population, which is easier to explain in the main text. One of your following questions also addresses this point.

Perhaps this was missed by the reviewer, but I did not see key words, are they required?

Our key words are phonocardiogram, heart murmur, health equity, PhysioNet Challenge, data science competition. We apologize for not including our keywords in the manuscript document.

A limitation of this paper is that the sample was not clearly described. The authors state that the PCG recording were from pediatric patients, yet pregnant individuals were mentioned as having contributed recordings. One might assume if pregnant women were included the PCG recording were obtained from a fetus on the abdomen of the mother? Among pediatric patients one might assume the PCG recordings were obtain from the child's chest? How might this impact recordings (chest vs abdomen) is this is how the PCG was recorded?

We agree that the patient population is not clearly described. The patient population is primarily pediatric, but it also includes pregnant adults. It does not include the fetuses of the pregnant patients.

We have clarified the description in the Challenge data section by changing:

> The CirCor DigiScope dataset [6] was used for the 2022 George B. Moody PhysioNet Challenge. This dataset consists of one or more PCG recordings from several auscultation locations.

to

> The CirCor DigiScope dataset [6] was used for the George B. Moody PhysioNet Challenge 2022. This dataset consists of 5268 PCG recordings from one or more auscultation locations during 1568 patient encounters with 1452 distinct patients. The patient population was primarily pediatric, ranging from neonates to adolescents, but it also included pregnant adults; no recordings were collected from fetuses.

The PCGs were collected from the chest in both the pregnant and non-pregnant individuals in the areas of the following valves: the pulmonary valve, aortic valve, mitral valve, and tricuspid valve; please see Fig. 1 of the manuscript..

While it may be outside of the scope of this paper one would also imagine that PCG recordings are impacted by heart rate, which in pediatric patients (in your study you included Neonate (birth to 27 days), Infant (28 days to 1 year), Child (1 to 11 years), Adolescent (12 to 18 years), or fetus, in the case of pregnant individuals, which would be very challenging since heart rate is so fast in younger children. Was heart rate considered or accounted for? Again, this may not be appropriate to discuss in this paper, but could be mentioned.

We agree that the higher heart rates in younger patients may make it more difficult to interpret their recordings. Many algorithms used age and heart rate as features, and some algorithms tried to segment the recordings, which could help to address this issue.

In general, there are many factors that may make it easier or harder to interpret the recordings. To encourage a diversity of approaches, and we intentionally do not identify these factors or suggest how to address them. We have added the following text to mention this issue:

> Some issues, such as background noises in the recordings and the relatively high heart rates of children, are inherent to the data or to the collection of the data. To preserve the realism of the problem and the diversity of the approaches, we did not remove these issues, enumerate them as potential difficulties, or suggest how to address them; many of the solutions employ different strategies to address different problems with the data.

In the Introduction, second sentence the term “technology is used. Yet a stethoscope is a device, which may be a more appropriate term.

The word “technology” referred to the act of auscultation with a stethoscope, not to the stethoscope itself. We have replaced “technology” with “tool” to avoid potential confusion.

Introduction second paragraph – the statements regarding “within the valves” is not entirely accurate. Suggest using “around,” rather than “within” as blood flow is disrupted around an incompetent valve. The authors may want to delete the statement “…..and by the turbulence of blood flow within the valves” in the first sentence and end it after “….cardiac cycle.” Then make the suggested adjustment in the next sentence with refers to the pathology.

We have updated and clarified the description of heart sounds in the Introduction by changing

> As acoustic signals, heart sounds are mainly generated by the vibrations of cardiac valves as they open and close during the cardiac cycle and by the turbulence of blood flow within the valves. Turbulent blood flow may cause enough vibrations within the cardiac valves to create audible heart sounds and abnormal waveforms in the PCG, which are called *murmurs*.

to

> As acoustic signals, heart sounds are mainly generated by the vibrations of cardiac valves as they open and close during the cardiac cycle. Turbulent blood flow may cause vibrations around the cardiac valves that create audible heart sounds and abnormal waveforms in the PCG, which are called *murmurs*.

Figure 1 – the AV and PV locations are both at the location of the second intercostal space yet the image shows them slightly off, suggesting that the PV is in the third intercostal space.

We have updated the figure to more clearly and accurately show the auscultation locations.

Page 4 second paragraph – Consider adding (described below) to the sentence “Each patient’s PCGs and clinical notes were also annotated for murmurs.” This addition would help the reader understand that this description will be discussed further down in the paper.

We have added “(described below)” to this sentence as suggested.

Can “resource constrained” be defined i.e., rural vs urban, socioeconomic etc. this was not entirely clear.

This is a very good question. We specifically considered cardiac screening capacity, primarily for pediatric heart disease, as a specific resource constraint. This capacity requires both clinical experts and clinical infrastructure, which, as you know, are themselves affected by population density, socioeconomic factors, geography, politics, education, culture, history, etc.

Our co-authors who serve this region shaped the specific problem and evaluation metric to address cardiac screening capacity as a specific resource constraint.

In general, the Challenges encourage the development of automated approaches that can perform as well, or better than, experts, with a goal of increasing accessibility to high-quality healthcare. The specific factors that may limit accessibility often vary, but the practices that we employ to improve accessibility remain similar from year to year.

We have addressed this observation by changing

> However, while cardiac auscultation itself is relatively accessible, experts are needed to interpret heart sounds and PCGs, limiting the accessibility of auscultation for cardiac disease management, especially in resource-constrained environments.

to

> However, while cardiac auscultation itself is relatively accessible, experts are needed to interpret heart sounds and PCGs, limiting the accessibility of auscultation for cardiac disease management, especially in resource-constrained environments, where limited access to clinical experts and infrastructure prevents widespread screening.

Page 6 sentence that reads “………………important issue in the Section .” seem to be missing the section #.

The journal’s LaTeX template does not include section numbers, so the \ref{example-section-label} command was blank. We have changed “...in the Section .” to “... in the Discussion section.” to address this mistake.

For the text description for Figures 3 and 4 consider bolding the text for “per-patient” and “per-screening” to add clarity to the description.

We have bolded both “per-patient” and “per-screening” as suggested.
